# Safety guidelines for youth agricultural work in the United States: A description of the development and updating process

**DOI:** 10.3389/fpubh.2023.1048718

**Published:** 2023-04-18

**Authors:** Andrea V. R. Swenson, Marsha Salzwedel, Cassandra Peltier, Barbara C. Lee

**Affiliations:** National Children’s Center for Rural and Agricultural Health and Safety, Marshfield Clinic Research Institute, Marshfield, WI, United States

**Keywords:** youth, work, guidelines, agriculture, intervention development

## Abstract

To reduce the prevalence of youth injuries and fatalities in agricultural settings, safety professionals considered developing a guideline-focused intervention for how and when youth should conduct farm chores. In 1996, the process to create guidelines started, which then expanded to include professionals from the United States, Canada, and Mexico. This team used a consensus driven approach to develop the guidelines and launch the North American Guidelines for Children’s Agricultural Tasks. By 2015, research related to the published guidelines indicated a need to incorporate new empirical evidence and develop dissemination plans based on new technologies. The process for updating the guidelines was supported by a 16-person steering committee and used content experts and technical advisors. The process yielded updated and new guidelines, now called Agricultural Youth Work Guidelines. This report responds to request for further details on the development and update of the guidelines and describes the genesis of the guidelines as an intervention, the process for creating guidelines, recognition of the need to update guidelines based on research, and the process for updating guidelines to assist in others engaged in similar types of interventions.

## 1. Introduction

In the late 1980s, an informal group of farm safety advocates began discussing various approaches for addressing the high frequency of serious injuries and fatalities of children across the more than 2 million agricultural operations in the United States. At that time, there were no public- or private-sector organizations dealing with safety and health specific to children living, working, or visiting farms. Since then, child safety advocates and stakeholders representing farm families worked together to develop an intervention aimed to prevent child injuries and fatalities within agricultural settings. Since the launching of the original guidelines in 1999, there have been requests for a description of the process used to create the guidelines. The updating of guidelines renewed calls for a thorough description of the process. Individuals have requested the description to reference the process used to create the guidelines for individuals implementing and conducting research with the guidelines and replicate the process in other health and safety topics. This report describes the genesis of the guidelines as an intervention, the process for creating guidelines, recognition of the need to update guidelines based on research, and the process for updating guidelines to assist in others engaged in similar types of interventions.

## 2. North American Guidelines for Children’s Agricultural Tasks

The development of the North American Guidelines for Children’s Agricultural Tasks (NAGCAT), which later became Agricultural Youth Work Guidelines (AYWG), started in the late 1980s. To address the prevalence of youth injuries and fatalities in agricultural settings, safety professionals considered developing recommendations for how and when youth should conduct farm chores. However, farm organization representatives expressed concern and skepticism regarding written guidelines. Further, pediatricians insisted there was no “wiggle room” for children to be present in any occupational setting. Given the spectrum of perspectives on guidelines, standards or protocols, it was evident that any recommendations required a consensus process that included all stakeholder groups.

In 1996, a formal proposal for creating working guidelines for children in agriculture was submitted within a larger grant application for an agricultural safety center of excellence funded by the National Institute for Occupational Safety and Health (NIOSH). A small level of funding was secured to form a working group, led by a core team in Marshfield, WI, United States. While initially planned to take about 1 year to complete, the multidisciplinary team grew, and the complexity of creating work guidelines became daunting.

Substantial funds for project expansion came with the establishment of the NIOSH-funded National Children’s Center for Rural and Agricultural Health and Safety (NCCRAHS) in 1997. The three-member core team established in 1996 was joined by 12 external advisors from the United States, Canada, and Mexico. Twice-yearly in-person meetings were scheduled for a 2-year endeavor. Inclusion/exclusion criteria for activity and documentation parameters were established. A definition and process was set for what constituted group “consensus” such as requiring three-fourths of the team members’ agreement. Early in the process, advisors with expertise in child development made a convincing argument for using child development principles (physical, social, intellectual, and emotional abilities) versus age as demarcations for when a child is capable of completing a task. At the same time, several risk managers insisted that work tasks be described using the job-hazard-analysis framework commonly used across industries (see U.S. Department of Labor, *Job Hazard Analysis* (1) for more information). The full team including all advisors agreed upon these strategies, setting the stage for a new approach to recommending guidelines for children’s work in agriculture.

The next question was “what are the most important jobs or tasks for which guidelines are needed?” Two strategies provided answers to this question. First, existing data sets such as state-level farm fatality reports revealed most common agents of injury to children. Second, a questionnaire sent to state-based farm safety specialists and members of the National Institute for Farm Safety, provided insights on what types of jobs and tasks were conducted by youth in their region. Data from these two sources yielded a list of more than 50 tasks that merited attention. An advisor with marketing expertise in agricultural personal protective equipment (PPE) recommended that the end-product be more than a series of charts and words, so visual depictions of potential printed resources for farm parents were reviewed. The team agreed an illustrator versus a photographer should be employed to convey key components of safety guidelines such as youth engaged in tasks as well as images of various types of PPE to be worn during work activities.

With the basic principles and project timeline established, work began in earnest. The team split up responsibilities. Most of the team was developing the content for each of the work tasks. This started by filling in the job-hazard-analysis framework based on how a task was conducted and often warranted a subsequent review by a producer active in that type of work. For example, for “feeding milk to calves” an advisor drafted the content, then a dairy farmer with children reviewed the step-by-step process, of filling and transporting a filled bottle to a calf hutch, holding the calf, supervising the youth, etc. Once the job-hazard-analysis framework was completed, the individual steps were reviewed by child development specialists to match tasks and hazards with required developmental characteristics of a child, addressing issues such as weight bearing, fatigue, required judgment, and supervision. This team also oversaw the drafting and finalization of each task illustration as well as the layout of how the full-color guidelines would eventually appear. Meanwhile, other team members addressed project management including meetings, documentation, budgets, timelines, and evaluation. By 1998, the guidelines were named the “North American Guidelines for Children’s Agricultural Tasks” or NAGCAT.

An overriding issue was preparing the guidelines for acceptance, dissemination, and adoption in the farming community. In the United States, one of the largest and most influential farm organizations is the American Farm Bureau Federation (AFBF; 2). The AFBF is one of many farm organizations that resist adding regulations affecting family farm practices, including parents’ rights to engage children in work. Thus, a pivotal step in handling any adverse reaction to the release of guidelines led to an in-person presentation to the AFBF Policy Committee. Although committee members questioned the need for guidelines and had major reservations that, over time, they would become regulations, they voted unanimously to “not take a public position” regarding NAGCAT. The team now felt confident in moving ahead with a highly public announcement about forthcoming guidelines.

*Successful Farming* magazine was given exclusive advance notice regarding the guidelines and prepared a centerfold fully illustrated story including farm parent interviews, timed with their actual completion. Nearly 200,000 reprints of the 12-page SF article were widely distributed across the United States and Canada. In addition, the National Institute for Farm Safety approved an opening 1999 conference session on NAGCAT that featured the most well-known farm radio broadcaster, Orion Samuelson, along with the National FFA Youth President. Both speakers expressed unabashed endorsement of NAGCAT as a resource intended to match a child’s ability with the risks and hazards of a farm task. The tagline of “helping kids do the job safely” conveyed the value of gaining important job skills.

**Figure 1:**
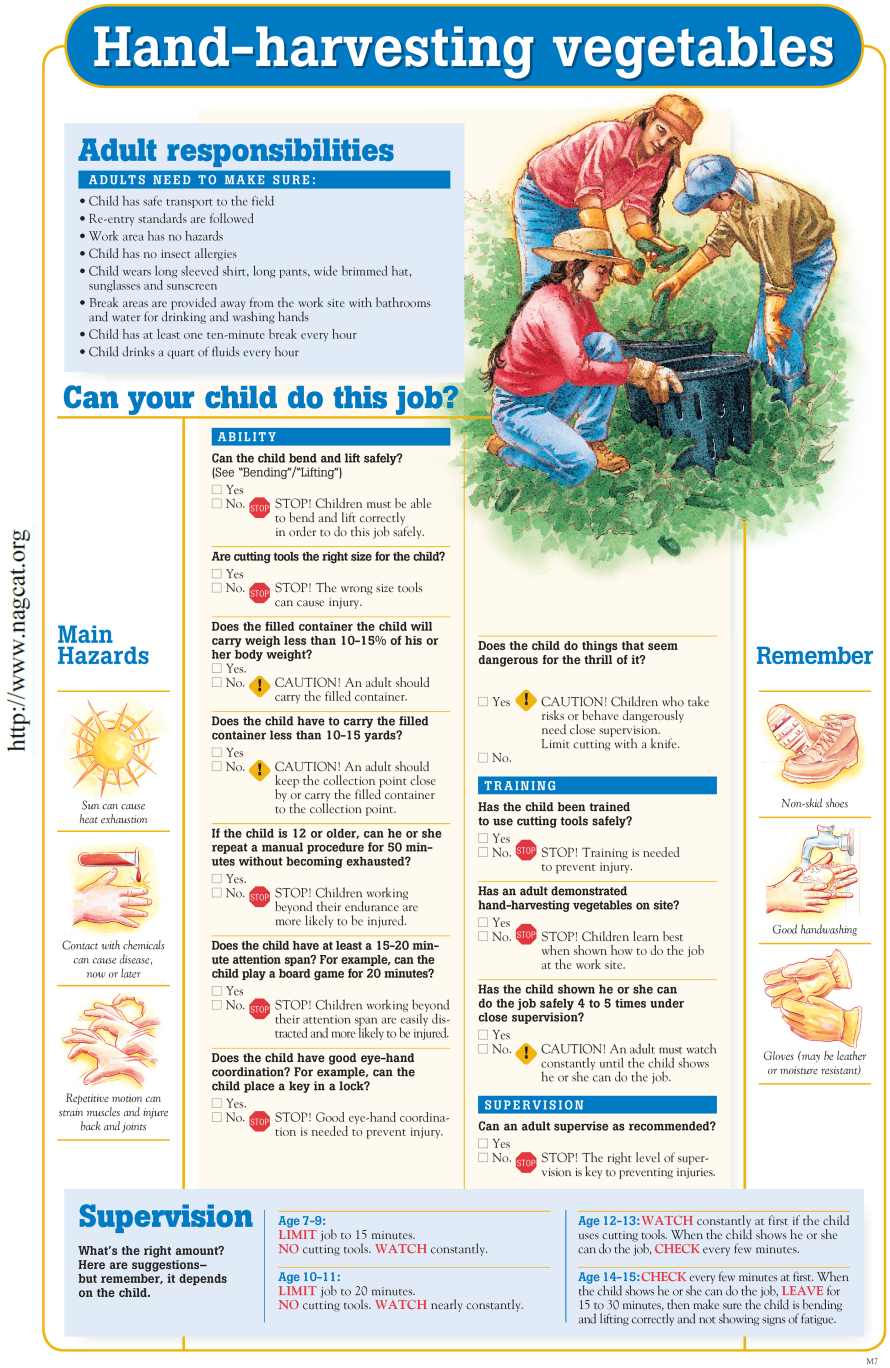
NAGCAT poster for hand-harvesting vegetables. Published with permission from the National Children’s Center for Rural and Agricultural Health and Safety.

The resulting product of 3 years’ of consensus-derived guidelines was a Professional Resource Binder with details of each job-hazard-analysis, the child development assessment for each job, and related materials. For the farming public, the resources were illustrated posters for each job. A full-color poster contained: (a) an illustration of a child conducting a task correctly; (b) a list of adult responsibilities; (c) main hazards of the task; (d) “things to remember”; and (e) a series of questions about “can my child do this job?” The summary statement recommended the amount of supervision needed based on an age range, noting, “remember, it depends upon the child.” In all, 62 specific and four general (e.g., “bending”) guidelines were released (see Figure 1). Topics ranged from detasseling corn to working with poultry and operating a tractor with an implement. A supplemental Tractor Operation matrix correlated tractor features (e.g., horsepower and implements) with a lower age range for youth. Guidelines were available to download from the internet and they were printed then bound into six categories, available for purchase from a safety supply vendor. Select guidelines were also available in Spanish and French.

Once farm safety specialists became more familiar with the guidelines, related resources were created, such as a video introduction for presentations to parents and a teacher’s manual for classroom use. Guidelines were endorsed by the International Labor Organization (ILO) and adapted for use in Sweden, Australia, and among the Hmong population in the Midwest US (3). Because the guidelines were created out of necessity, without a substantial empirical basis, over the next decade NIOSH prioritized research on the validity of guideline content, efficacy of guidelines when applied and related activities.

## 3. Research findings and need for updating NAGCAT

Reviews of research related to NAGCAT are presented in Marlenga, Lee, and Pickett’s 2012 article on implications for the future (4) as well as Doty and Marlenga’s 2006 article outlining priorities for the future of NAGCAT (5). Research examined the content of NAGCAT (tractors, jobs involving lifting and carrying, and supervision), dissemination strategies, and efficacy of preventing injuries. Findings indicate the use of NAGCAT could prevent serious injuries (6), including a case–control study that revealed a 50% reduction of work-related injuries for tasks where a guideline was applicable on farms in upstate New York (7).

Implications from research included a need to continue to incorporate new empirical evidence into the guidelines and develop dissemination plans (4). These needs continued to become more evident with emerging scientific evidence on requirements to safely perform work tasks as well as technology’s influence on how work was accomplished on farms (8) and how farm parents and supervisors accessed information (9). In late 2015, the illustrated, paper-printed guidelines were available *via* a website, but users needed to download the poster from the website and print it out for reference. A digital version that was mobile responsive to various electronic devices was increasingly important. Finally, although some NAGCAT were adapted to address needs of different cultural populations, it was recognized that some populations did not readily identify with the guidelines (e.g., illustrations only depicted white children) and were inaccessible due to language barriers (10–12). After discussion with key stakeholders, NCCRAHS adopted a strategy to make a “second edition” or “upgraded model,” including a mobile-friendly update with relevant content changes, rather than make small changes to select jobs at frequent intervals.

## 4. Process for updating NAGCAT

National Children’s Center for Rural and Agricultural Health and Safety began the process of updating NAGCAT in 2016. Funding was provided through NIOSH, with supplemental funding from CHS Community Giving. The objectives for the process were to account for the following items while updating guidelines: (a) evidence-based recommendations for activities and issues germane to child development (physical, social, intellectual, and emotional); (b) current child ag injury/fatality data; (c) changes in production agriculture; (d) proposed changes in child labor regulations; (e) lessons learned about the consensus development process; (f) information technology and health communications theory/practice; (g) updated recommendations for adults; and (h) priority topics. To ensure these objectives were achieved, a steering committee of 16 stakeholders across agricultural industries (e.g., farmers, American Farm Bureau, USDA, NIOSH, and Progressive Ag Foundation) was formed. The steering committee provided overall guidance on topics, assessed practicality versus science issues, focused messaging, addressed cultural relevancy, and guided overall design/format.

**Figure 2:**
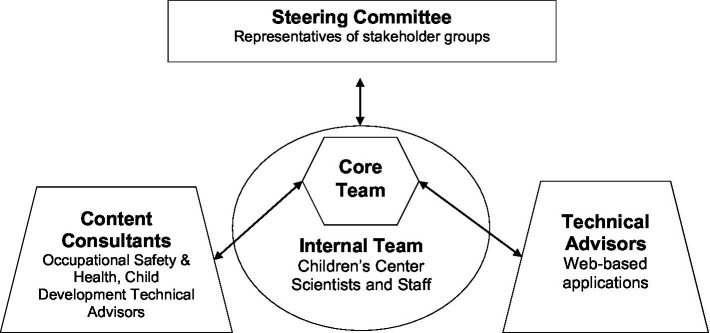
Overview of team relationships.

National Children’s Center for Rural and Agricultural Health and Safety staff formed an internal team who were responsible for helping create initial drafts/content and planning processes, facilitated relationships/communication with other projects, helped review content, and assisted with promotion and agricultural health and safety researchers, information technology specialists, graphic designers, and media relations specialists. A three-person core team was responsible for the day-to-day activities of the project and met weekly to manage the project’s content, technology, communications, and collaborations. The core team worked closely with content consultants who provided expertise in occupational safety and health and child development, and technical advisors who provided expertise in web-based applications. Figure 2 provides an overview of the relationships between different teams involved in the update process.

Updating the guidelines started with a review of 10 tasks (e.g., driving a farm tractor, unloading grain, and bending). The Steering Committee reviewed the list and provided feedback on the topics as well as input on additional tasks young workers were engaged in on farms. Potential content consultants were solicited from the Steering Committee and Internal Team. Once confirmed, these consultants were given the original job hazard analysis framework and a checklist for a task and were asked to update its content using the latest scientific evidence. Fresh content was developed for newly added guidelines. Once completed, the job hazard analysis framework and checklist was reviewed by a child development specialist who identified developmental concerns. These materials were brought to the Steering Committee and the Internal Team to update guidelines and generate the new guidelines for topics decided upon by the Steering Committee. Examples of new topics included milking cows in a dairy parlor, operating a lawn mower, operating a utility task vehicle, operating an unmanned aerial vehicle (i.e., drone), refueling equipment, and working outdoors.

**Figure 3:**
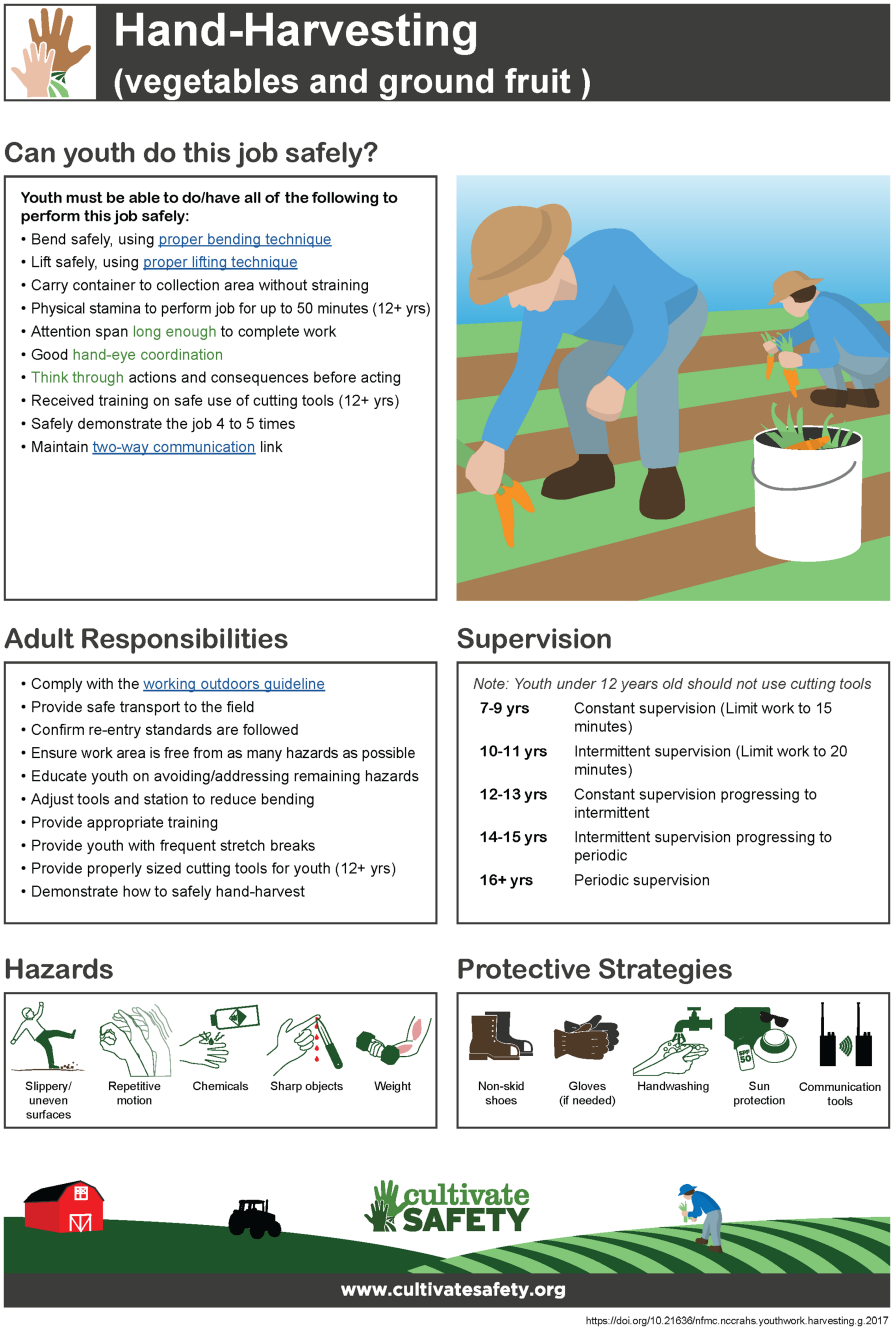
AYWG for hand-harvesting. Green text in the guideline provides pop-up definitions while blue underlined text links to supplemental materials. Published with permission from the National Children’s Center for Rural and Agricultural Health and Safety.

Lead by the Core Team, the Steering Committee, Internal Team, and Technical Team worked to create the format of the new resources. The decision was made to rename the North American Guidelines for Children’s Agricultural Tasks to Agricultural Youth Work Guidelines (AYWG). The format of guidelines was updated to include what the youth needs to be able to do to perform the task safely, adult responsibilities, supervision, and the most common hazards and protective strategies (see Figure 3). Guidelines were hosted on a website, www.cultivatesafety.org/aywg, and available in read, print, download, and interactive forms. Each guideline displays a graphic of a person performing the task, which can be customized for skin tone (light, medium, and dark) and equipment color (red, orange, yellow, green, and blue). The online guidelines link to other relative information (e.g., connect/disconnect an implement links to a page on bending, lifting, and climbing safety) and uses tooltips to provide definitions for important terms/concepts (e.g., mature and peripheral vision). Hazards and protective strategies have visuals associated with each item listed to aid in comprehension of the dangers and how to protect oneself. All guidelines are available in English, French, and Spanish. The guidelines are available on a mobile-friendly website to enable easy access to the guidelines in multiple formats (interactive, view, print, and download) from a smartphone or tablet, thus, increasing their utility in the work setting.

Following the creation of AYWG, the Core Team developed several supplemental materials to aid in their use and dissemination. These include content on the benefits of farm work, supervision, child development, communication, and bending, lifting, and climbing. Three topic-based booklets were developed—Safety Guidelines for Youth Operating Farm Equipment, Safety Guidelines for Youth Working in Gardens, and Safety Guidelines for Youth Working with Animals. Each booklet contains 18–31 related guidelines, information on how to best use the guidelines, and related resources. The booklets are available in English, French, and Spanish in both digital and hard copy formats. A media kit was also created with guidance from the Steering Committee and shared through the website. Table 1 provides an overview of the key differences between the original guidelines and the updated version.

**Table 1:** Comparison of work guidelines.

	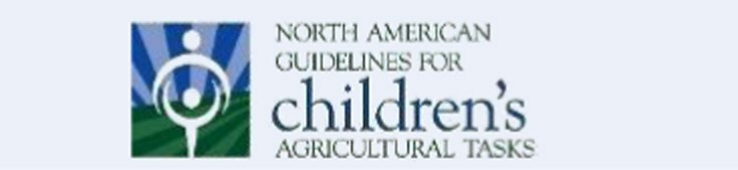	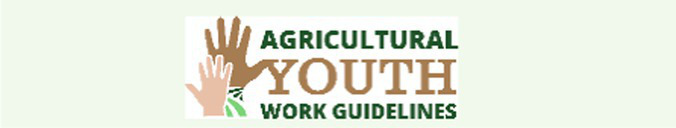
Acronym	NAGCAT	AYWG
Release date	1999	Final updates released in 2020
Development Process	Consensus of 30 safety professionals; series of in-person meetings, teleconferences, e-communications; and producer consultants	Sixteen-person steering committee; series of in-person and teleconference meetings; use of job hazard analysis frameworks from NAGCAT and published research; expert content consultants
Poster Format	Paper, PDF	Paper, PDF, Read, Print, and Interact
Graphics	Illustrated Drawings	Vector Graphics
PDF Features		Tooltips, Hyperlinks
Website	Static	Mobile responsive
Customizable	None	Skin tones, Equipment colors
Number	62 + 5 Supplemental Tractor guidelines outlining cognitive, physical, etc. development	48 + 5 Supplemental Tractor guidelines outlining cognitive, physical, etc. development
Language	English +10 in Spanish	English, French, and Spanish
Supplemental Materials	Resource Manual: Job hazard analyses charts; child development checklists; training materials; and calendars	Job hazard analysis charts, checklists, bending, lifting & climbing videos, and factsheet; Supervision, communication, child development, and benefits of farm work
Booklets	Two booklets: Farm equipment, gardening	Three booklets: Farm Equipment, Gardening, Animals

## 5. Current initiatives and next steps

The new guidelines were debuted in the opening keynote session of the 2017 International Society for Agricultural Safety and Health. Since their debut, AYWG have been and continue to be featured in numerous presentations, webinars, press releases, newsletter articles, social media posts, and other activities. Dissemination was accelerated in response to the COVID-19 pandemic. With more school-aged children present on farms due to school closings and the cancelation of extra-curricular events, organizations and media turned to the AYWG as a way to help parents keep children safe on farms. For example, the COVID-19 Interim Guidance from the Centers for Disease Control and Prevention (CDC) and the U.S. Department of Labor for Agriculture Workers and Employers provided links to the AYWG to address concerns of assignment of age-appropriate tasks to children on farms (13).

The newest initiatives involve dissemination and implementation research to guide future activities and, ultimately, increase the adoption of AYWG into practice. By incorporating evidence-based dissemination and implementation strategies, practitioners will be able to more effectively distribute materials and information to farm parents and youth supervisors to ultimately prevent agricultural injuries and fatalities in youth. Part of this process is to assess the needs of the target populations, including Latinx-owned farms and educators. Two studies currently underway investigate factors influencing the use of AYWG among farming populations and youth educators. Findings from these studies will develop recommendations for reducing barriers and increasing motivators for using the guidelines, recommendations for messaging for organizations and end-users, and suggested modifications for future versions of AYWG.

Agricultural Youth Work Guidelines are the only known intervention aimed at reducing risk to injury and fatalities for youth working in agriculture by assessing the match between what agricultural tasks require and youth capabilities. This report describes the process of guideline generation from reconnection of a need, developing the intervention to address the need, and the process of updating the intervention. Transparency in how the guidelines were created and updated will provide researchers and practitioners valuable information as they work with guidelines and interventions in the future.

## Data availability statement

The original contributions presented in the study are included in the article/supplementary material, further inquiries can be directed to the corresponding author/s.

## Author contributions

All authors listed have made a substantial, direct, and intellectual contribution to the work and approved it for publication.

## Funding

Primary funding for the National Children’s Center for Rural and Agricultural Health and Safety—National Institute for Occupational Safety and Health—U54OH009568.

## Conflict of interest

The authors declare that the research was conducted in the absence of any commercial or financial relationships that could be construed as a potential conflict of interest.

## Publisher’s note

All claims expressed in this article are solely those of the authors and do not necessarily represent those of their affiliated organizations, or those of the publisher, the editors and the reviewers. Any product that may be evaluated in this article, or claim that may be made by its manufacturer, is not guaranteed or endorsed by the publisher.
